# When Benign Turns Critical: Surgical Management of an Asymptomatic Renal Angiomyolipoma With Inferior Vena Cava and Right Cardiac Extension

**DOI:** 10.7759/cureus.90539

**Published:** 2025-08-19

**Authors:** Jiehua Li, Lunchang Wang, Xin Li, Hao He, Chang Shu

**Affiliations:** 1 Department of Vascular Surgery, The Second Xiangya Hospital of Central South University, Changsha, CHN; 2 Institute of Vascular Diseases, Central South University, Changsha, CHN; 3 Center of Vascular Surgery, Fuwai Hospital, National Center for Cardiovascular Disease, Chinese Academy of Medical Sciences and Peking Union Medical College, Beijing, CHN

**Keywords:** inferior vena cava extension, renal angiomyolipoma, right atrial tumor thrombus, surgical management, vascular invasion

## Abstract

Renal angiomyolipoma (AML) is a benign mesenchymal tumor usually confined to the kidney, but rare cases exhibit aggressive behavior with extension into the inferior vena cava (IVC) and right atrium (RA), resulting in significant clinical challenges due to risks such as heart failure and pulmonary embolism. We report a case of a 42-year-old asymptomatic woman with right renal AML demonstrating progressive vascular invasion from the kidney to the IVC and RA over two years. The computed tomography (CT) scan revealed a fat-containing tumor with IVC and RA extension. Surgical management comprised right nephrectomy combined with tumor thrombectomy under cardiopulmonary bypass. The patient underwent successful radical resection without perioperative complications. Histopathological analysis confirmed AML, and postoperative follow-up showed no recurrence or residual tumor, with preserved renal function. This case underscores the rare but critical presentation of renal AML with vascular and cardiac extension, emphasizing the importance of comprehensive imaging for diagnosis and surgical planning. Despite its complexity, radical surgery offers favorable outcomes and is the preferred treatment to prevent life-threatening complications. Vigilant monitoring remains essential for timely intervention in similar cases.

## Introduction

Renal angiomyolipoma (AML) is a rare benign mesenchymal tumor of the kidney comprising variable amounts of adipose tissue, smooth muscle, and abnormal blood vessels [1]. It is often detected incidentally during imaging studies performed for unrelated indications, with most lesions remaining asymptomatic and confined to the kidney. Clinical manifestations, when present, typically relate to tumor size and may include flank pain, hematuria, or a palpable abdominal mass [2]. A critical clinical concern for AML is spontaneous hemorrhage due to fragile vasculature within the tumor, which can lead to retroperitoneal bleeding and hemodynamic instability.

While AML generally exhibits benign behavior, extremely rare cases have been reported with tumor thrombus extension into the inferior vena cava (IVC) and right atrium (RA), posing unique diagnostic and therapeutic challenges [3]. Such vascular and cardiac involvement significantly increases risks of cardiovascular complications, including right heart failure and pulmonary embolism, necessitating timely recognition and intervention [4,5].

Imaging modalities such as ultrasonography, computed tomography (CT), and magnetic resonance imaging (MRI) are essential in detecting and delineating the extent of AML, particularly when vascular invasion is suspected. Fat-containing lesions can usually be identified on these imaging platforms; however, "fat-poor" variants require careful differential diagnosis to exclude malignant renal neoplasms.

Given the rarity and clinical significance of renal AML with IVC and cardiac extension, here, we present a case highlighting the diagnostic approach and surgical management strategy for this uncommon but potentially life-threatening presentation.

## Case presentation

A 42-year-old woman was referred to our tertiary care center for further evaluation of a right renal mass with inferior vena cava (IVC) extension, initially detected incidentally on abdominal ultrasound two years prior. Surveillance imaging revealed progressive tumor growth, with recent ultrasonography demonstrating extension into the right atrium (RA). Notably, the patient remained entirely asymptomatic, denying constitutional symptoms (fever, weight loss), cardiorespiratory complaints (dyspnea, chest pain, hemoptysis, or palpitations), or flank discomfort. Her medical history included an uncomplicated caesarean section 17 years earlier and extracorporeal shock wave lithotripsy for a right-sided ureteral stone one month before admission. She reported no family history of renal tumors, tuberous sclerosis, or other genetic disorders.

Physical examination revealed a hemodynamically stable patient (blood pressure: 135/88 mmHg; pulse: 76 bpm; respiratory rate: 20/min; afebrile at 36.5°C). Cardiopulmonary auscultation was unremarkable, with no murmurs, rubs, or gallops. Abdominal examination showed no tenderness, organomegaly, or palpable masses. There was no evidence of lower extremity edema or signs of deep vein thrombosis.

Laboratory investigations were unremarkable: complete blood count, renal function (serum creatinine:56.0 μmol/L; blood urea nitrogen: 5.19 μmol/L), and electrolytes were within normal limits. Inflammatory markers (C-reactive protein: 1.58 mg/L; procalcitonin: <0.05 ng/mL; erythrocyte sedimentation rate: 7 mm/h) and coagulation profiles (International normalized ratio: 0.98; D-dimer: 0.11 µg/mL FEU) showed no abnormalities. Tumor markers (CEA, AFP, CA125, CA19-9) were negative. Laboratory results at the time of admission are shown in Table 1.

**Table 1:** Laboratory results at the time of admission.

Test	Result	Reference range
White blood cell	7.01	3.50-9.50 10^9/L
Hemoglobin	136	115-150 g/L
Platelet count	197	125-350 10^9/L
Blood urea nitrogen	5.19	2.90-7.14 mmol/L
Creatinine	56.0	44.0-133.0 µmol/L
Sodium	137.2	137.0-147.0 mmol/L
Potassium	4.28	3.50-5.30 mmol/L
N-terminal pro-B-type natriuretic peptide	28.2	0.0-125.0 pg/mL
Troponin T	<3.000	0.00-14.0 pg/mL
Albumin	37.8	40.0-55.0 g/L
Prothrombin time (PT)	11.2	10.0-14.0 s
Fibrinogen	2.1	2.0-4.0 g/L
International normalized ratio (INR)	0.98	0.85-1.20
D-dimer	0.11	0.00-0.55 µg/mL FEU
Erythrocyte sedimentation rate (ESR)	7	0-20 mm/h
C-reactive protein	1.58	0.00-6.00 mg/L
Procalcitonin	<0.050	0.000-0.050 ng/mL
Carcinoembryonic antigen (CEA)	0.65	0.00-5.00 ng/mL
Alpha-fetoprotein (AFP)	1.34	0.00-20.00 ng/mL
CA-125	15.28	0.00-35.00 U/mL
CA19-9	0.81	0.00-35.00 U/mL

**Figure 1:**
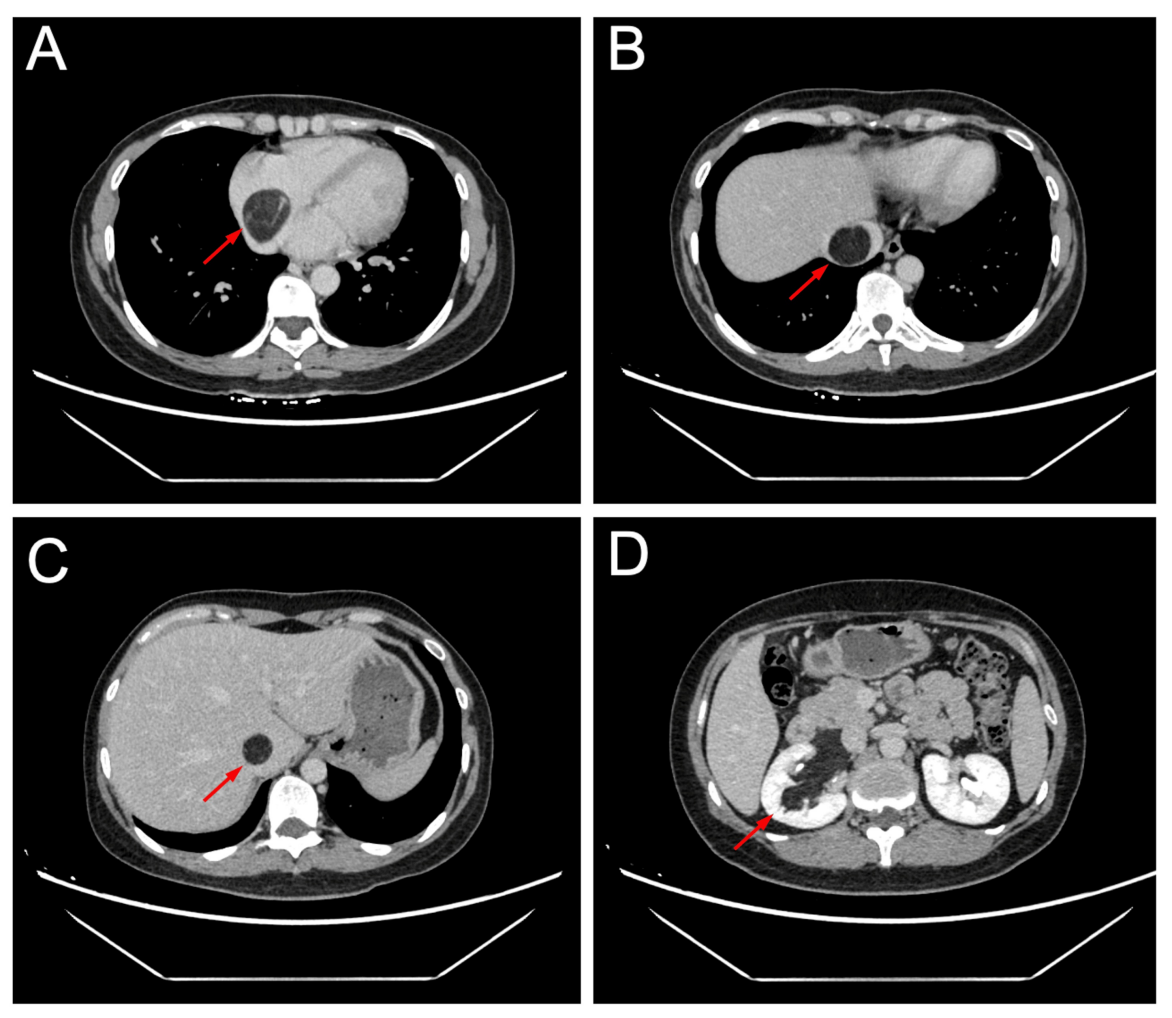
Axial views of the chest and abdominal computed tomography (CT) scan of the patient on admission. The axial views of CT scan showing hypodense masses in the right atrium (A), inferior vena cava (IVC) (B, C), and right kidney (D). (Arrows indicated the masses)

**Figure 2:**
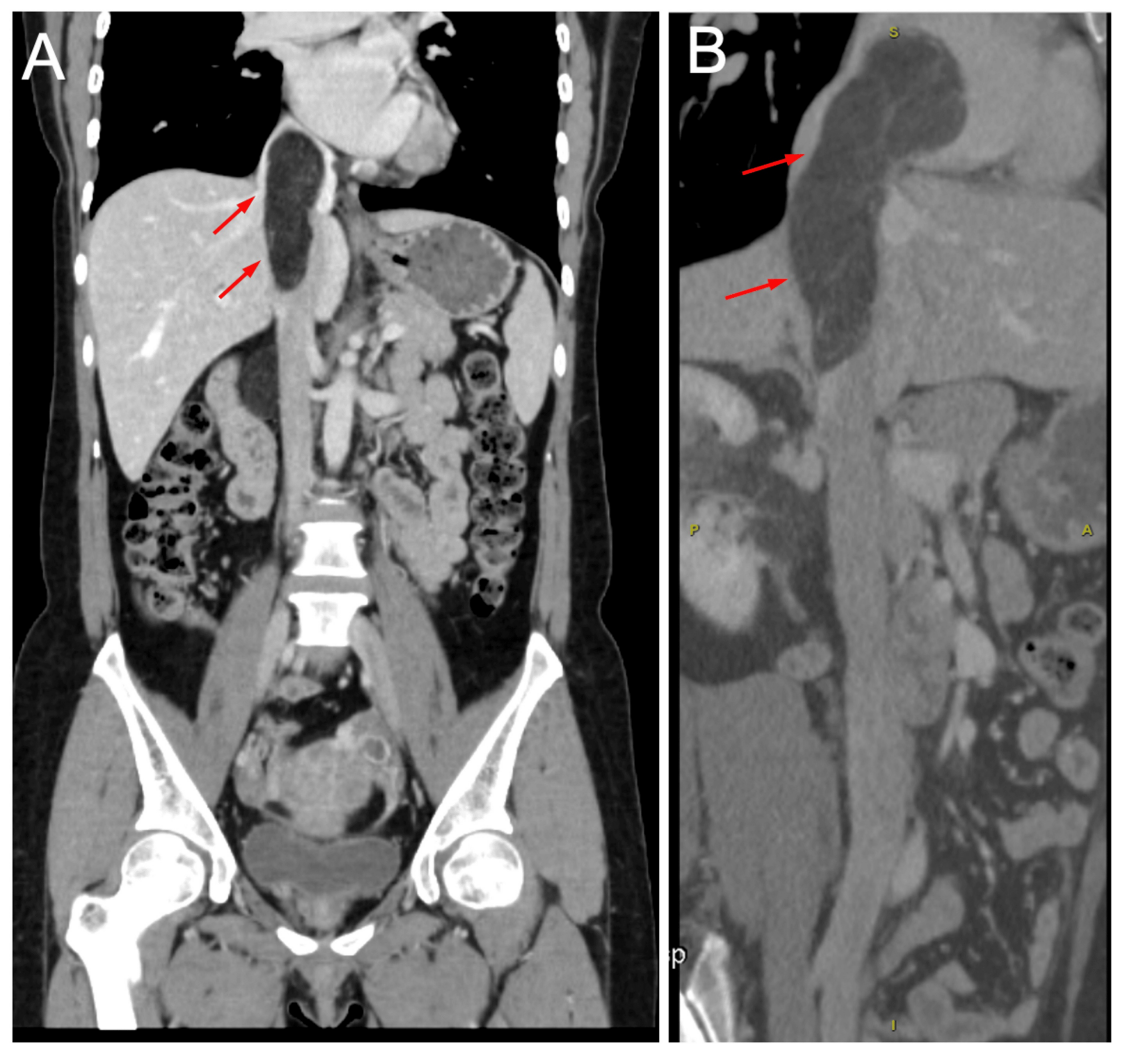
Coronal and two-dimensional reconstruction views of the chest and abdominal computed tomography (CT) scan of the patient on admission. (A) Coronal view of CT scan showing a hypodense tumor within the inferior vena cava (IVC). (B) Two-dimensional reconstruction view of the CT scan showing the hypodense tumor in IVC with right cardiac extension.

The computed tomography (CT) of the chest and abdomen revealed a well-circumscribed, fat-density mass (60 × 26 × 48 mm) arising from the right kidney, with a contiguous tumor thrombus extending into the IVC and right atrium (30 × 26 × 82 mm) (Figures 1, 2). No pulmonary embolism or lymphadenopathy was observed. The transthoracic echocardiography showed preserved biventricular function (ejection fraction: 60%) without valvular abnormalities.

**Figure 3:**
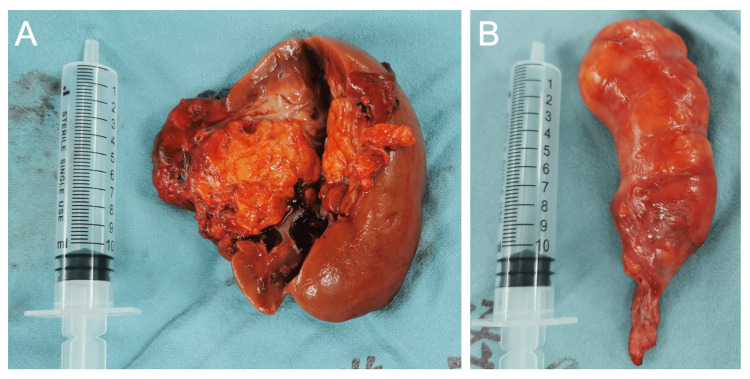
Gross specimen of the right kedney and tumor. (A) Gross specimen indicating the tumor originating from the kidney. (B) Gross specimen showing the tumor with a cylindrical shape and fatty color.

**Figure 4:**
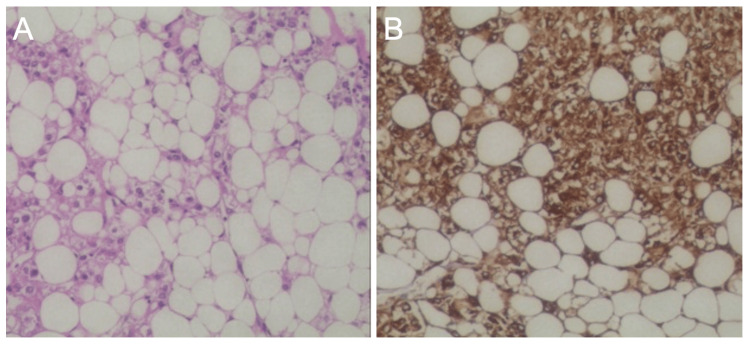
Pathological study of the tumor confirms the diagnosis of renal angiomyolipoma. (A) H&E staining shows the tumor consists of adipose tissue, smooth muscle cells and vascular wall tissue. (B) Immunohistochemical study shows positive staining of Melan-A.

Multidisciplinary management involving urology, cardiovascular surgery, vascular surgery, and anesthesiology was pursued due to the tumor’s complexity. The patient underwent en bloc resection via a combined laparotomy and median sternotomy approach. Intraoperative transesophageal echocardiography-guided tumor extraction under cardiopulmonary bypass. The specimen demonstrated macroscopic features typical of AML: a cylindrical, yellowish mass with a greasy cut surface (Figure 3). Histopathological examination revealed a triphasic morphology-mature adipose tissue, thick-walled blood vessels, and smooth muscle bundles-with immunohistochemical positivity for Melan-A and smooth muscle actin (SMA), confirming renal AML (Figure 4).

**Figure 5:**
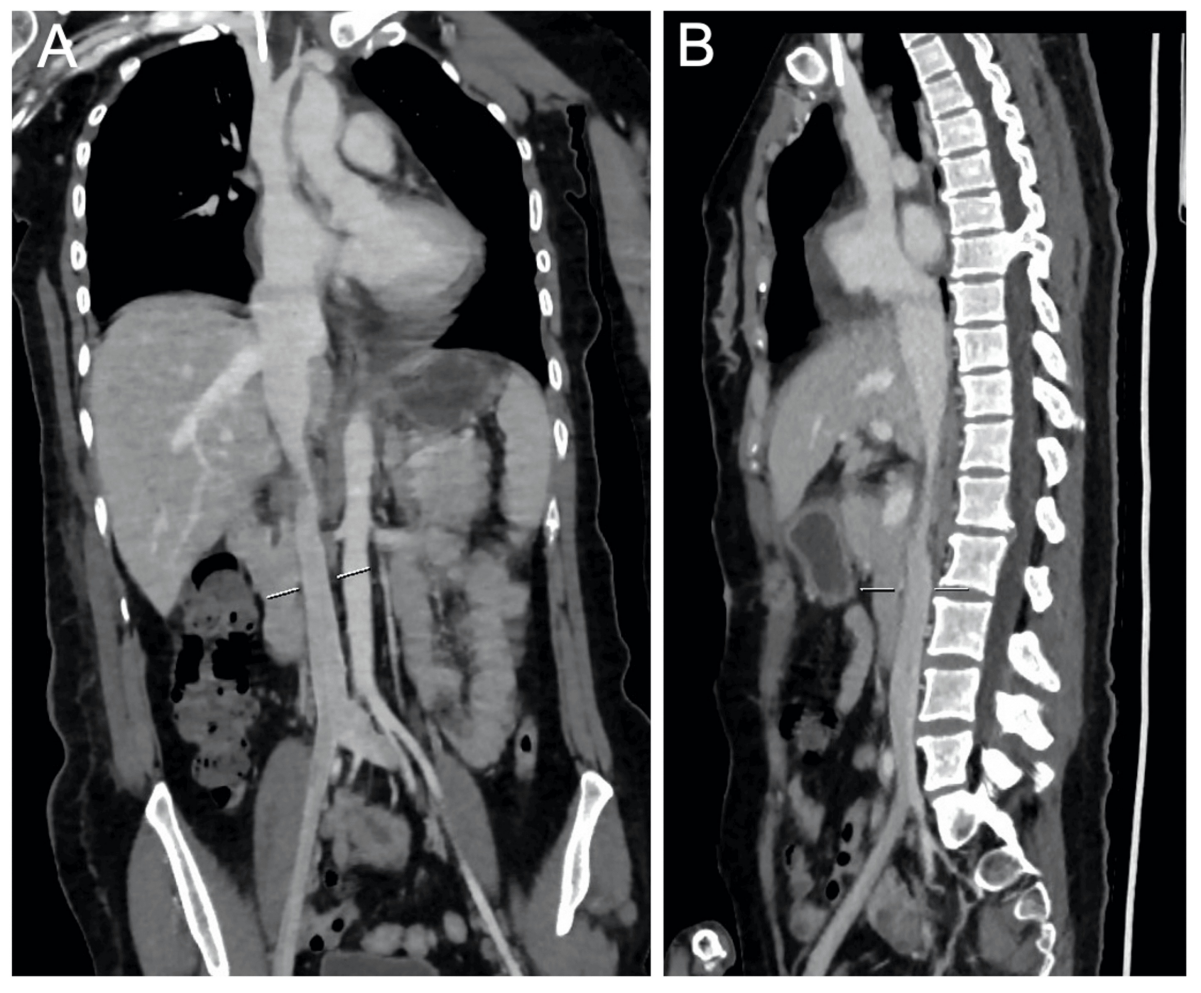
The seven-day post-operative CT scan shows the total extraction of the tumor. (A) The coronal view of post-operative CT scan shows total extraction of the tumor. (B) The sagittal view of post-operative CT shows no residual tumors.

**Figure 6:**
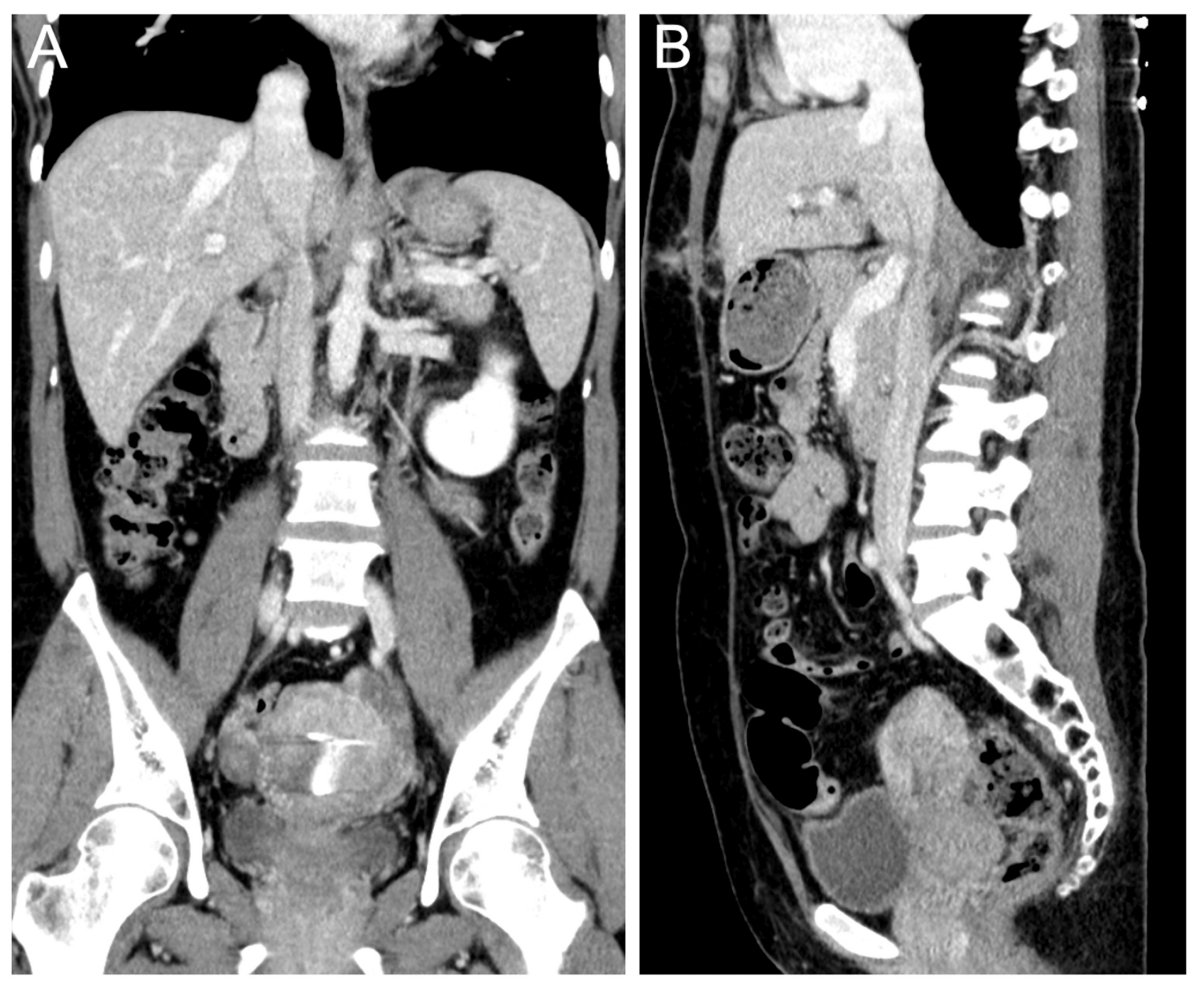
The six-month follow-up CT scan shows no recurrent tumors. (A) The coronal view of the follow-up CT scan shows no recurrent tumors. (B) The sagittal view of the follow-up CT shows no recurrent tumors.

Postoperative course was uneventful. A seven-day follow-up CT confirmed complete tumor resection (Figure 5), and surveillance imaging at six months demonstrated IVC patency without recurrence (Figure 6). The patient remained asymptomatic at her two-year follow-up, with no evidence of metastatic disease or local relapse, and her renal function remained normal.

## Discussion

Renal AML is a benign hamartomatous tumor of the kidney characterized histologically by the presence of three distinct components: adipose tissue, smooth muscle, and abnormal blood vessels. Although relatively rare, with an estimated incidence of approximately 0.13% in the general population, renal AML predominantly affects middle-aged women and may occur either sporadically (80%) or in association with tuberous sclerosis complex (TSC) (20%) [6,7]. The typical clinical course of renal AML is benign and indolent, but its behavior can be variable depending on multiple factors including tumor size, involvement of adjacent structures, and genetic context.

The vast majority of renal AMLs remain confined to the kidney without aggressive behavior. Nonetheless, a small subset of cases, particularly those with extensive involvement of the inferior vena cava (IVC) and even extension into the right atrium of the heart, present a unique diagnostic and therapeutic challenge due to their rarity and potentially life-threatening complications [8,9]. This manifestation is uncommon but clinically significant because tumor thrombus extending into the IVC and cardiac chambers can lead to severe cardiovascular complications such as right heart failure, obstruction of venous return, and fatal pulmonary embolism.

Renal AML usually remains clinically silent and is often discovered incidentally on imaging performed for unrelated reasons. When symptoms do occur, they frequently correlate with tumor size and include flank pain, hematuria, or palpable mass; these are more common in tumors exceeding 4 cm in diameter [10]. Larger lesions are also prone to spontaneous hemorrhage due to fragile, abnormal intratumoral vasculature, resulting in perinephric hematoma or retroperitoneal hemorrhage, which can cause hypovolemic shock and necessitate urgent medical intervention [11]. The presence of IVC and right cardiac extension further exacerbates the risk profile and may be associated with signs of vascular obstruction or embolic phenomena.

Accurate diagnosis of renal AML - especially with vascular extension - relies on a combination of clinical, radiological, and histopathological evaluations. Ultrasonography, computed tomography (CT), and magnetic resonance imaging (MRI) are pivotal in both diagnosing the tumor and determining its extent [12]. On ultrasound, AML appears as a markedly echogenic (bright) renal mass due to its high fat content. CT scans provide further diagnostic specificity by revealing areas of fat attenuation (−10 Hounsfield units or lower), which is a hallmark of AML. MRI can augment this evaluation through fat suppression sequences that highlight fat-containing lesions, aiding in differentiation from other renal masses. However, a diagnostic challenge arises in approximately 5% of AMLs that lack detectable fat, referred to as “fat-poor” AMLs. These lesions may mimic malignant renal neoplasms such as renal cell carcinoma, necessitating careful differential diagnosis to avoid misclassification [13]. Differential diagnoses in the context of IVC and cardiac involvement include intravenous leiomyomatosis (a benign smooth muscle proliferation), vascular leiomyosarcoma (a rare malignant smooth muscle tumor), thrombus formation secondary to renal vein or IVC thrombosis, renal cell carcinoma with tumor thrombus, lipoma, liposarcoma, teratoma, and adrenal myelolipoma. Comprehensive imaging combined with histopathologic confirmation via biopsy or surgical specimen analysis is therefore essential.

The exact mechanisms underlying vascular and cardiac extension of renal AML remain not fully understood, but several hypotheses exist. The neoplastic vasculature within AML is typically thin-walled and dysplastic, predisposed to rupture and intravascular growth. Tumor cells may invade renal veins and progressively extend along venous structures into the IVC and right atrium. The aggressive intravascular growth seen in these rare cases may reflect biological heterogeneity among AMLs, possibly influenced by genetic factors such as those associated with TSC mutations, which can promote abnormal cellular proliferation and adhesion. In addition, the invasion of large veins and atrial structures can facilitate tumor embolism, a serious and potentially fatal complication. Reported cases of renal AML causing pulmonary fat embolism underscore the clinical urgency to address tumor thrombus promptly to prevent embolic events [14,15].

Therapeutic decisions for renal AML depend largely on tumor size, symptoms, and presence of complications including hemorrhage or vascular extension [16]. For small, asymptomatic AMLs, conservative management with active surveillance is often appropriate. Selective arterial embolization (SAE) has emerged as a minimally invasive option to control tumor growth and bleeding risk, particularly in patients who are poor surgical candidates or have bilateral/multifocal lesions. However, renal AML complicated by tumor thrombus extending into the IVC and right heart generally necessitates radical surgical intervention due to the high risks of heart failure and pulmonary embolism. Surgery typically involves nephrectomy, either partial or total, alongside thrombectomy to remove the intravascular tumor component. When the tumor extends into the cardiac chambers, thoracotomy with cardiopulmonary bypass is required to facilitate complete tumor excision safely. Surgical treatment poses significant technical challenges owing to the need for multidisciplinary coordination among urologists, vascular surgeons, and cardiac surgeons, as well as intraoperative management of potential massive bleeding and hemodynamic instability. Nevertheless, reported outcomes indicate that patients who undergo complete resection of renal AML with vascular and cardiac involvement have favorable prognoses with low recurrence rates [17,18].

In the absence of malignant transformation, renal AML generally has an excellent prognosis following appropriate treatment. For patients with extensive vascular involvement, early diagnosis and radical surgical management are crucial for survival and functional recovery. Postoperative follow-up includes routine imaging to monitor for tumor recurrence or residual thrombus and assessment of renal function. The risk of recurrence after complete tumor and thrombus removal appears low, but long-term data are limited due to the rarity of such cases. For AML patients with TSC, vigilant surveillance is especially important because of the tendency toward bilateral and multifocal lesions, as well as possible rapid tumor growth [19]. In addition, the advent of mammalian target of rapamycin (mTOR) inhibitors, such as everolimus, has provided a pharmacologic modality to shrink AMLs by targeting the aberrant cell growth pathways associated with TSC, opening new therapeutic avenues for complex or inoperable cases [20].

Despite advances in imaging and surgical techniques, the rarity of renal AML with IVC and cardiac extension limits large-scale studies, and most data are derived from case reports or small case series. Consequently, standardized protocols for diagnosis, treatment, and follow-up have not been universally established. Further studies are needed to elucidate the molecular characteristics that predispose some AMLs to aggressive vascular invasion and to optimize minimally invasive or pharmacologic therapies. In addition, research into early biomarkers and noninvasive imaging modalities to distinguish fat-poor AMLs from malignant renal tumors could improve diagnostic accuracy and reduce unnecessary surgeries. The potential roles of targeted therapies in preventing tumor thrombus formation or recurrence also warrant investigation.

## Conclusions

Renal AML is a benign renal neoplasm and typically following an indolent course. However, the subset of AMLs that extend into the IVC and right heart represent a high-risk entity requiring meticulous diagnostic evaluation and aggressive surgical management. Multimodal imaging is essential for accurate diagnosis and surgical planning, while radical nephrectomy combined with thrombectomy and cardiopulmonary bypass for cardiac involvement offers satisfactory outcomes. The prognosis after successful tumor resection is generally favorable, though long-term vigilance is necessary to detect recurrence or manage related complications. Advancements in understanding tumor biology and the application of targeted therapies hold promise for improving care in these complex cases. 
